# 2,2′-(4-Amino-4*H*-1,2,4-triazole-3,5-di­yl)diphenol

**DOI:** 10.1107/S1600536809055536

**Published:** 2010-01-09

**Authors:** Sheng-Hui Chen, Gao-Yong Zhang, Jin-Feng Dong

**Affiliations:** a College of Chemistry and Molecular Sciences, Wuhan University, Wuhan 430072, People’s Republic of China

## Abstract

The structure of the title compound, C_14_H_12_N_4_O_2_, was determined as part of a project on the coordination chemistry of 1,2,4-triazole derivatives. In the crystal structure, one of the two benzene rings is almost coplanar with the five-membered triazole ring (mean deviation = 0.019 Å), whereas the second benzene ring is rotated by 51.973 (2)°. The two N—C—N—N torsion angles [170.365 (2) and −170.942 (3)°] indicate that the amido group is slightly twisted away from the triazole plane. An intra­molecular O—H⋯N hydrogen bond occurs. In the crystal structure, inter­molecular N—H⋯O and O—H⋯N hydrogen bonding is found.

## Related literature

For background information on tthe coordination chemistry of 1,2,4-triazole derivatives, see: Lavrenova *et al.* (1995 ▶).
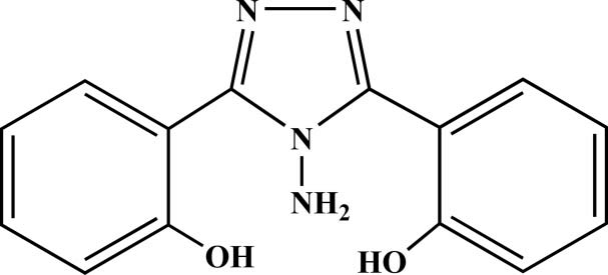


## Experimental

### 

#### Crystal data

C_14_H_12_N_4_O_2_
*M* *_r_* = 268.28
Orthorhombic, 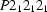

*a* = 8.262 (2) Å
*b* = 9.384 (3) Å
*c* = 15.919 (4) Å
*V* = 1234.2 (6) Å^3^
*Z* = 4
Mo *K*α radiation
μ = 0.10 mm^−1^
*T* = 296 K
0.10 × 0.10 × 0.08 mm

#### Data collection

Bruker SMART CCD area-detector diffractometer
Absorption correction: multi-scan (*SADABS*; Sheldrick, 1996 ▶) *T*_min_ = 0.980, *T*_max_ = 0.992
6343 measured reflections
1276 independent reflections
1143 reflections with *I* > 2σ(*I*)
*R*_int_ = 0.043

#### Refinement

*R*[*F* ^2^ > 2σ(*F* ^2^)] = 0.041
*wR*(*F* ^2^) = 0.112
*S* = 1.10
1276 reflections
192 parameters
H atoms treated by a mixture of independent and constrained refinement
Δρ_max_ = 0.18 e Å^−3^
Δρ_min_ = −0.17 e Å^−3^

### 

Data collection: *SMART* (Bruker, 1997 ▶); cell refinement: *SAINT* (Bruker, 1997 ▶); data reduction: *SAINT*; program(s) used to solve structure: *SHELXS97* (Sheldrick, 2008 ▶); program(s) used to refine structure: *SHELXL97* (Sheldrick, 2008 ▶); molecular graphics: *SHELXTL* (Sheldrick, 2008 ▶); software used to prepare material for publication: *SHELXTL*.

## Supplementary Material

Crystal structure: contains datablocks global, I. DOI: 10.1107/S1600536809055536/nc2167sup1.cif

Structure factors: contains datablocks I. DOI: 10.1107/S1600536809055536/nc2167Isup2.hkl

Additional supplementary materials:  crystallographic information; 3D view; checkCIF report

## Figures and Tables

**Table 1:** Hydrogen-bond geometry (Å, °)

*D*—H⋯*A*	*D*—H	H⋯*A*	*D*⋯*A*	*D*—H⋯*A*
N4—H4*A*⋯O1^i^	0.95 (5)	2.39 (6)	3.153 (4)	136 (4)
O1—H1⋯N1	0.82	1.87	2.598 (3)	148
O2—H2*A*⋯N2^ii^	0.82	1.91	2.705 (3)	162

Symmetry codes: (i) 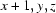; (ii) 
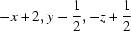
.

**Fig. 1.**
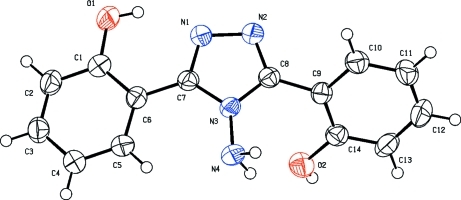
Crystal structure of the title compound showing the atom-labelling scheme. Displacement ellipsoids are drawn at the 50% probability level.

## supplementary crystallographic information

### Experimental

2-hydroxy-*N*-(2-hydroxybenzoyl)benzohydrazide and hydrazine monohydrate were purchased from Acros and used without further purification. 2-hydroxy-*N*-(2-hydroxybenzoyl)benzohydrazide (4.08 g, 0.15 mmol) and hydrazine monohydrate(7.27 ml, 0.15 mol) were transfered into a  50 ml round-bottom flask. The mixture was stirred and refluxed for 3 h. After cooling, the solution was poured into ice water and the resulting light pink precipitate was collected by filtration, and recrystallized from ethanol. colorless crystal, 1.812 g (46.6 %), ^1^H NMR (DMSO, 400 MHz, p.p.m.): 11.27 (s, 2H), 8.0 (q, 2H), 7.4 (m, 2H), 7.0 (q, 4H), 6.14 (s, 2H). m.p = 532.3–533.2 K. Anal. Calcd for C_14_H_12_O_2_N_4_: C, 62.69; H, 4.48; N, 20.90%, Found: C, 62.58; H, 4.42; N, 20.83 %. GC—MS(m/*z*): 268 (*M*^+^), 249, 221, 207, 117, 91, 77, 51, 32.

### Refinement

All C-H H atoms were placed in geometrically idealized positions (methyl H atoms  allowed to rotate but not to tip) and constrained to ride on their parent atoms with C—H distances in the range of 0.93–0.98 Å, and with *U*_iso_(H)  = 1.2 U _eq_ for aryl H atoms and 1.5 *U*_eq_ for the methyl H atoms. The O-H and N-H H atoms were located in difference map and were refined isotropic with varying coordinates. Because no strong anomalous scaterring atoms are present the absolute structure cannot be determined, Therefore, Friedel-opposites were merged in the refinement and the absolute structure was selected arbitrarily.

### Crystal data

**Table:**

C_14_H_12_N_4_O_2_	*F*(000) = 560
*M**_r_* = 268.28	*D*_x_ = 1.444 Mg m^−^^3^
Orthorhombic, *P*2_1_2_1_2_1_	Mo *K*α radiation, λ = 0.71073 Å
Hall symbol: P 2ac 2ab	Cell parameters from 2036 reflections
*a* = 8.262 (2) Å	θ = 2.4–24.6°
*b* = 9.384 (3) Å	µ = 0.10 mm^−^^1^
*c* = 15.919 (4) Å	*T* = 296 K
*V* = 1234.2 (6) Å^3^	Block, colorless
*Z* = 4	0.10 × 0.10 × 0.08 mm

### Data collection

**Table:**

Bruker SMART CCD area-detector diffractometer	1276 independent reflections
Radiation source: fine-focus sealed tube	1143 reflections with *I* > 2σ(*I*)
graphite	*R*_int_ = 0.043
phi and ω scans	θ_max_ = 25.0°, θ_min_ = 2.5°
Absorption correction: multi-scan (*SADABS*; Sheldrick, 1996)	*h* = −9→9
*T*_min_ = 0.980, *T*_max_ = 0.992	*k* = −9→11
6343 measured reflections	*l* = −18→16

### Refinement

**Table:**

Refinement on *F*^2^	Secondary atom site location: difference Fourier map
Least-squares matrix: full	Hydrogen site location: inferred from neighbouring sites
*R*[*F*^2^ > 2σ(*F*^2^)] = 0.041	H atoms treated by a mixture of independent and constrained refinement
*wR*(*F*^2^) = 0.112	*w* = 1/[σ^2^(*F*_o_^2^) + (0.0693*P*)^2^ + 0.078*P*] where *P* = (*F*_o_^2^ + 2*F*_c_^2^)/3
*S* = 1.10	(Δ/σ)_max_ = 0.005
1276 reflections	Δρ_max_ = 0.18 e Å^−^^3^
192 parameters	Δρ_min_ = −0.17 e Å^−^^3^
0 restraints	Extinction correction: *SHELXL97* (Sheldrick, 2008), Fc^*^=kFc[1+0.001xFc^2^λ^3^/sin(2θ)]^-1/4^
Primary atom site location: structure-invariant direct methods	Extinction coefficient: 0.011 (4)

### Special details

**Table:**

Geometry. All esds (except the esd in the dihedral angle between two l.s. planes) are estimated using the full covariance matrix. The cell esds are taken into account individually in the estimation of esds in distances, angles and torsion angles; correlations between esds in cell parameters are only used when they are defined by crystal symmetry. An approximate (isotropic) treatment of cell esds is used for estimating esds involving l.s. planes.
Refinement. Refinement of F^2^ against ALL reflections. The weighted R-factor wR and goodness of fit S are based on F^2^, conventional R-factors R are based on F, with F set to zero for negative F^2^. The threshold expression of F^2^ > 2sigma(F^2^) is used only for calculating R-factors(gt) etc. and is not relevant to the choice of reflections for refinement. R-factors based on F^2^ are statistically about twice as large as those based on F, and R- factors based on ALL data will be even larger.

### Fractional atomic coordinates and isotropic or equivalent isotropic displacement parameters (Å^2^)

**Table:**

	*x*	*y*	*z*	*U*_iso_*/*U*_eq_	
C1	0.5404 (4)	0.5286 (4)	0.49447 (17)	0.0385 (7)	
C2	0.4539 (4)	0.4330 (4)	0.54359 (19)	0.0449 (8)	
H2	0.3416	0.4386	0.5450	0.054*	
C3	0.5310 (4)	0.3309 (4)	0.5899 (2)	0.0457 (8)	
H3	0.4711	0.2671	0.6220	0.055*	
C4	0.6981 (4)	0.3225 (4)	0.5890 (2)	0.0479 (8)	
H4	0.7509	0.2536	0.6208	0.057*	
C5	0.7861 (4)	0.4166 (3)	0.5408 (2)	0.0442 (8)	
H5	0.8985	0.4103	0.5406	0.053*	
C6	0.7108 (4)	0.5211 (3)	0.49212 (17)	0.0339 (7)	
C7	0.7987 (4)	0.6149 (3)	0.43411 (18)	0.0339 (7)	
C8	0.9790 (3)	0.7037 (3)	0.34798 (17)	0.0333 (7)	
C9	1.1311 (3)	0.7234 (3)	0.30083 (17)	0.0345 (7)	
C10	1.1826 (4)	0.8612 (3)	0.2823 (2)	0.0422 (8)	
H10	1.1286	0.9388	0.3055	0.051*	
C11	1.3126 (4)	0.8838 (4)	0.2298 (2)	0.0521 (9)	
H11	1.3482	0.9759	0.2186	0.062*	
C12	1.3895 (4)	0.7686 (4)	0.1941 (2)	0.0586 (10)	
H12	1.4744	0.7837	0.1568	0.070*	
C13	1.3427 (4)	0.6316 (4)	0.2127 (2)	0.0482 (9)	
H13	1.3981	0.5550	0.1894	0.058*	
C14	1.2130 (4)	0.6075 (3)	0.26611 (18)	0.0371 (7)	
N1	0.7263 (3)	0.7099 (3)	0.38646 (15)	0.0388 (6)	
N2	0.8404 (3)	0.7657 (3)	0.33109 (15)	0.0395 (6)	
N3	0.9594 (3)	0.6108 (3)	0.41328 (15)	0.0335 (6)	
N4	1.0844 (3)	0.5384 (4)	0.45696 (19)	0.0427 (7)	
O1	0.4530 (3)	0.6247 (3)	0.44960 (15)	0.0554 (7)	
H1	0.5143	0.6734	0.4210	0.083*	
O2	1.1615 (3)	0.4745 (2)	0.28606 (13)	0.0458 (6)	
H2A	1.1785	0.4206	0.2465	0.069*	
H4A	1.167 (7)	0.605 (5)	0.471 (3)	0.097 (16)*	
H4B	1.131 (5)	0.486 (4)	0.419 (2)	0.059 (12)*	

### Atomic displacement parameters (Å^2^)

**Table:**

	*U*^11^	*U*^22^	*U*^33^	*U*^12^	*U*^13^	*U*^23^
C1	0.0354 (17)	0.0409 (17)	0.0393 (15)	0.0028 (14)	−0.0007 (13)	−0.0023 (14)
C2	0.0329 (17)	0.052 (2)	0.0500 (17)	−0.0040 (15)	0.0028 (15)	0.0022 (16)
C3	0.0440 (19)	0.0430 (19)	0.0500 (18)	−0.0045 (16)	0.0092 (16)	0.0025 (15)
C4	0.0438 (18)	0.047 (2)	0.0531 (18)	0.0030 (16)	0.0074 (15)	0.0123 (16)
C5	0.0367 (17)	0.0430 (19)	0.0529 (18)	0.0052 (15)	0.0008 (14)	0.0087 (16)
C6	0.0321 (16)	0.0312 (16)	0.0385 (15)	−0.0029 (13)	0.0013 (12)	−0.0051 (13)
C7	0.0317 (15)	0.0291 (16)	0.0408 (15)	0.0000 (14)	0.0011 (12)	−0.0003 (13)
C8	0.0326 (15)	0.0280 (15)	0.0391 (14)	−0.0011 (13)	−0.0007 (12)	−0.0023 (13)
C9	0.0310 (15)	0.0344 (17)	0.0382 (15)	−0.0025 (13)	−0.0014 (12)	−0.0009 (13)
C10	0.0392 (17)	0.0343 (17)	0.0530 (18)	−0.0042 (14)	−0.0030 (15)	0.0003 (15)
C11	0.0391 (17)	0.046 (2)	0.071 (2)	−0.0101 (18)	−0.0009 (17)	0.0159 (18)
C12	0.0346 (18)	0.074 (3)	0.067 (2)	−0.0013 (19)	0.0118 (16)	0.013 (2)
C13	0.0326 (16)	0.057 (2)	0.0552 (19)	0.0087 (16)	0.0062 (15)	−0.0016 (18)
C14	0.0336 (15)	0.0377 (17)	0.0400 (15)	0.0003 (14)	−0.0047 (13)	0.0016 (14)
N1	0.0342 (13)	0.0348 (15)	0.0474 (13)	0.0007 (12)	0.0030 (11)	0.0056 (12)
N2	0.0368 (14)	0.0341 (14)	0.0477 (14)	0.0021 (11)	0.0044 (11)	0.0045 (12)
N3	0.0263 (12)	0.0335 (13)	0.0409 (12)	0.0003 (11)	0.0003 (10)	0.0009 (11)
N4	0.0313 (15)	0.0460 (17)	0.0507 (17)	0.0061 (13)	−0.0032 (12)	0.0065 (15)
O1	0.0302 (12)	0.0693 (18)	0.0666 (15)	0.0058 (12)	0.0032 (11)	0.0211 (14)
O2	0.0577 (15)	0.0305 (12)	0.0493 (12)	−0.0016 (11)	0.0076 (11)	−0.0071 (10)

### Geometric parameters (Å, °)

**Table:**

C1—O1	1.358 (4)	C9—C10	1.393 (4)
C1—C2	1.388 (4)	C9—C14	1.395 (4)
C1—C6	1.410 (5)	C10—C11	1.377 (5)
C2—C3	1.366 (5)	C10—H10	0.9300
C2—H2	0.9300	C11—C12	1.376 (5)
C3—C4	1.383 (5)	C11—H11	0.9300
C3—H3	0.9300	C12—C13	1.375 (5)
C4—C5	1.377 (5)	C12—H12	0.9300
C4—H4	0.9300	C13—C14	1.386 (4)
C5—C6	1.396 (4)	C13—H13	0.9300
C5—H5	0.9300	C14—O2	1.356 (4)
C6—C7	1.468 (4)	N1—N2	1.393 (3)
C7—N1	1.314 (4)	N3—N4	1.419 (3)
C7—N3	1.368 (4)	N4—H4A	0.95 (5)
C8—N2	1.312 (4)	N4—H4B	0.87 (4)
C8—N3	1.366 (4)	O1—H1	0.8200
C8—C9	1.475 (4)	O2—H2A	0.8200
			
O1—C1—C2	116.8 (3)	C14—C9—C8	121.2 (3)
O1—C1—C6	123.4 (3)	C11—C10—C9	120.6 (3)
C2—C1—C6	119.8 (3)	C11—C10—H10	119.7
C3—C2—C1	121.1 (3)	C9—C10—H10	119.7
C3—C2—H2	119.4	C12—C11—C10	119.3 (3)
C1—C2—H2	119.4	C12—C11—H11	120.3
C2—C3—C4	120.0 (3)	C10—C11—H11	120.3
C2—C3—H3	120.0	C13—C12—C11	121.0 (3)
C4—C3—H3	120.0	C13—C12—H12	119.5
C5—C4—C3	119.8 (3)	C11—C12—H12	119.5
C5—C4—H4	120.1	C12—C13—C14	120.1 (3)
C3—C4—H4	120.1	C12—C13—H13	119.9
C4—C5—C6	121.6 (3)	C14—C13—H13	119.9
C4—C5—H5	119.2	O2—C14—C13	122.4 (3)
C6—C5—H5	119.2	O2—C14—C9	118.2 (3)
C5—C6—C1	117.7 (3)	C13—C14—C9	119.4 (3)
C5—C6—C7	123.3 (3)	C7—N1—N2	108.2 (2)
C1—C6—C7	118.7 (3)	C8—N2—N1	107.1 (2)
N1—C7—N3	108.7 (2)	C8—N3—C7	106.4 (2)
N1—C7—C6	123.0 (3)	C8—N3—N4	126.3 (2)
N3—C7—C6	128.0 (3)	C7—N3—N4	126.9 (2)
N2—C8—N3	109.6 (3)	N3—N4—H4A	109 (3)
N2—C8—C9	125.7 (3)	N3—N4—H4B	105 (3)
N3—C8—C9	124.6 (3)	H4A—N4—H4B	103 (4)
C10—C9—C14	119.4 (3)	C1—O1—H1	109.5
C10—C9—C8	119.0 (3)	C14—O2—H2A	109.5

### Hydrogen-bond geometry (Å, °)

**Table:**

*D*—H···*A*	*D*—H	H···*A*	*D*···*A*	*D*—H···*A*
N4—H4A···O1^i^	0.95 (5)	2.39 (6)	3.153 (4)	136 (4)
O1—H1···N1	0.82	1.87	2.598 (3)	148
O2—H2A···N2^ii^	0.82	1.91	2.705 (3)	162

Symmetry codes: (i) *x*+1, *y*, *z*; (ii) −*x*+2, *y*−1/2, −*z*+1/2.
